# The importance of BMPs and TGF-βs for endochondral bone repair – A longitudinal study in hip arthroplasty patients

**DOI:** 10.1016/j.bonr.2023.101723

**Published:** 2023-11-10

**Authors:** Jean Cassuto, Agnetha Folestad, Jan Göthlin, Henrik Malchau, Johan Kärrholm

**Affiliations:** a Orthopedic Research Unit & Department of Orthopedic Surgery, Sahlgrenska University Hospital, Mölndal, Sweden; b Department of Orthopedics, CapioLundby Hospital, Göteborg, Sweden; c Department of Radiology, Sahlgrenska University Hospital, Mölndal, Sweden; d Institution of Clinical Sciences, Göteborg University, Göteborg, Sweden; e Department of Orthopedic Surgery, Harvard Medical School, Boston, USA

**Keywords:** Bone regeneration, Transforming growth factor beta, Bone morphogenic proteins, Hip arthroplasty

## Abstract

**Introduction:**

Osseointegration of hip implants, although a decade-long process, shows striking similarities with the four major phases of endochondral bone repair. In the current study we investigated the spatiotemporal involvement of bone morphogenic proteins (BMPs) and transforming growth factor betas (TGF-βs) throughout the process of bone repair leading to successfully osseointegrated hip implants.

**Materials and methods:**

Twenty-four patients that had undergone primary total hip arthroplasty (THA) due to one-sided osteoarthritis (OA) were investigated during a period of 18 years (Y) with repeated measurements of plasma biomarkers as well as clinical and radiological variables. All implants were clinically and radiographically well anchored throughout the follow-up. Eighty-one healthy donors divided in three gender- and age-matched groups and twenty OA patients awaiting THA, served as controls. Plasma was analyzed for BMP-1, -2, -3, -4, -6, -7 -9 and TGF-β1, -β2, -β3 by use of a high-sensitivity and wide dynamic range electrochemiluminescence technique allowing for detection of minor changes.

**Results:**

Spatiotemporal changes during the follow-up are presented in the context of the four phases of endochondral bone repair shown in earlier studies and transposed to the current study based on similarities in biomarker responses. Phase 1: *Primary proinflammatory phase* lasting from surgery until day 7, Phase 2: *Chondrogenic phase* from day 7 until 18 months postsurgery, Phase 3: *Secondary proinflammatory and cartilage remodeling phase* lasting from 18 months until 7Y, Phase 4: *coupled bone remodeling* from 7Y until 18Y postsurgery. BMP-1 increased sharply shortly after surgery and remained significantly above healthy during the chondrocyte recruitment, proliferation, and hypertrophy phases with a subsequent return to control level at 5Y postsurgery. BMP-2 was above healthy controls before surgery and 1 day after surgery before decreasing to control level and remaining there throughout the follow-up. BMP-3 was at control level from presurgery until 6M after surgery when it increased to a peak at 2Y during the cartilage hypertrophy phase followed by a gradual decrease to control level at 10Y during the phase of bone formation. In the following, BMP-3 decreased below controls to a nadir 15Y postsurgery during coupled bone remodeling. BMP-4 was at control level from presurgery until 10Y postsurgery when it increased to a sharp peak at 15Y after surgery followed by a return to the level of healthy at 18Y. BMP-6 did not differ from healthy during the follow-up. BMP-7 was at control level from presurgery until 1Y postsurgery before gradually increasing to a peak at 10Y during the early phase of osteogenesis with a gradual return to control level at 18Y during the phase of coupled bone remodeling. BMP-9 was above OA before surgery followed by a decrease to basal level on day 1 after surgery and a renewed increase to a plateau above controls lasting from 6 W until returning to the level of healthy at 18Y postsurgery, i.e., throughout the phases of cartilage formation, cartilage hypertrophy and remodeling, bone formation and coupled bone remodeling. TGF-β1 was above controls presurgery before decreasing to baseline shortly after surgery followed by a renewed increase at 6 M to a peak at 2Y during cartilage hypertrophy/remodeling followed by a gradual return to baseline at 10Y during early osteoblastogenesis. TGF-β2 was at control level from presurgery until the phase of cartilage remodeling at 5Y when it increased sharply to a peak at 7Y with a gradual return to baseline at 18Y postsurgery. TGF-β3 remained at control level throughout the study.

**Conclusion:**

This study shows that the involvement of BMPs and TGF-βs in endochondral bone repair is a process of stepwise recruitment of individual biomarkers characterized by distinct, yet overlaping, spatiotemporal patterns that extend from the early phase of pre-chondrocyte recruitment until the late phase of coupled bone remodeling.

## Highlights

• The unique spatial and temporal pattern of BMPs during endochondral repair
• The distinctive spatiotemporal appearance of TGF-βs during endochondral repair
• The significance of individual BMPs and TGF-βs for endochondral bone healing
• Potential redundancies and crosstalk between BMPs and TGF-βs
• Points of interaction between BMPs, TGF-βs and other bone regulating pathways

## 1 Introduction

Extensive research has proven that bone morphogenic proteins (BMPs) and transforming growth factor betas (TGF-βs), both members of the TGF-β superfamily, are an integral part of skeletal development and fracture repair (Cho et al., 2002; Marsell and Einhorn, 2009; Poniatowski et al., 2015; Gerstenfeld et al., 2003). The TGF-β family is comprised of TGF-β1, -β2 and -β3 that, despite having a high degree of structural homology with common receptors and signaling pathways, show significant spatiotemporal and functional differences during bone repair (Cho et al., 2002; Marsell and Einhorn, 2009; Poniatowski et al., 2015; Gerstenfeld et al., 2003). TGF-βs are secreted and stored in the extracellular matrix (ECM) as latent inactive complexes of two dimerized TGF-β precursors and two latency associated proteins (LAPs) (Poniatowski et al., 2015). Activation of TGF-βs require proteolytic release from the ECM followed by cleavage of LAPs that enable the active molecules to form a complex with their transmembrane receptors, i.e., transforming growth factor beta receptor I, II and III (TGFβRI-III) and trigger intracellular signaling through Smad proteins that translocate to the nucleus and activate or inhibit target genes (Poniatowski et al., 2015). The abbreviation “smad” refers to the homologies of the *Caenorhabditis elegans* SMA (“small” worm phenotype) and MAD family (“Mothers Against Decapentaplegic”) of genes in Drosophila. Activation of TGF-βs can be executed by a variety of proteolytic enzymes and is highly regulated by several soluble and cell-anchored antagonists (Poniatowski et al., 2015). BMPs are, in similarity with TGF-βs, secreted as inactive precursors that need enzymatic activation before being able to attach to their surface receptors, i.e., bone morphogenic protein receptor I and II (BMPRI, BMPRII) and activin type II receptor (ActRII) thereby triggering intracellular smad signaling (Marsell and Einhorn, 2009). The activities of BMPs are controlled by intracellular inhibitors that target cytoplasmic smad signaling as well as by circulating antagonists that compete for the BMP receptors or attach to BMP ligands and prevent them from accessing their receptors (Marsell and Einhorn, 2009).

The current study is based on the same population of hip arthroplasty patients and healthy controls as two previous studies that monitored bone regulating biomolecules during a two-decade follow-up and discussed their role in the process of endochondral bone repair leading to a successful osseointegration of implants (Cassuto et al., 2018; Cassuto et al., 2020). The aim of the present study was to monitor the spatiotemporal patterns of individual BMPs and TGF-βs during a two-decade follow-up and discuss their potential role in the context of the different phases of endochondral repair previously reported (Gerstenfeld et al., 2003; Loi et al., 2016; Marsell and Einhorn, 2011; Einhorn and Gerstenfeld, 2015; Bahney et al., 2019).

## 2 Materials and methods

### 2.1 Study design and study population

The study, with level of evidence II, was approved by the institutional review board of VästraGötalandsRegionen at Sahlgrenska University Hospital and informed consent was obtained from every patient prior to inclusion. The study complies with the STROBE-statement for observational studies (von Elm et al., 2014). (I) Sixty consecutive patients scheduled for primary total hip arthroplasty (THA) due to osteoarthritis (OA) were enrolled into the study to evaluate different femoral stem designs with clinical parameters, radiography, radiostereometry (RSA) and dual-energy X-ray absorptiometry (DEXA) (Karrholm et al., 2002; Thien et al., 2012). In addition, venous blood was sampled on each visit. The THA group received either of two uncemented stems i.e., Epoch®, a low-modulus stem with porous coating and reduced stiffness (*n* = 10) and Anatomic®, a titanium alloy stem with porous coating (*n* = 14), both from Zimmer-Biomet, Warsaw, Indiana, USA. Both stem types were supplied with an additional layer of hydroxyapatite and tricalciumphosphate (HA/TCP) on the porous coating. The Anatomic stems were also coated with pure hydroxyapatite distal to the porous coating. All patients received a cementless porous press-fit cup with HA/TCP coating (Trilogy®, Zimmer-Biomet) on the acetabular side fixed with or without additional screws. All cups were supplied with polyethylene liners, gamma-sterilized with 0.025 Gy in inert nitrogen gas. Characteristics, such as wear rates, stem migration patterns, loss of BMD and clinical outcome, were reported for the implants in a 7-year follow-up (Thien et al., 2012). Basic demographic data, comorbidities and medications were registered. Patients with a pre-existing hip implant prior to the index implant (*n* = 14) were excluded. Patients who developed clinical (see HHS and pain scores) and/or radiological signs of OA in the opposite hip joint during the follow-up (*n* = 12), were excluded. Eight patients with lysis or implant wear prompting revision surgery and two patients with clear-cut lysis, but no revision, were excluded. Patients diagnosed with prosthesis loosening during the follow-up were not included in the current investigation as they proved to be too few to allow for a proper statistical evaluation. (I) A total of twenty-four patients (mean age 58Y, range 40–69; 16 males and 8 females) with one-sided total hip implant that had remained clinically and radiographically stable during a two-decade follow-up, with the contralateral hip not showing clinical or radiographic signs of OA, were included. (II) Twenty OA patients awaiting THA (mean age 58Y, range 34–86; fourteen males and 6 females), were included as a control of the stability and validity of biomarkers in pooled plasma. Each biomarker was assessed by comparing levels in plasma stored for 18Y (presurgery sample, PR) with fresh plasma from OA patients awaiting THA. (III) Eighty-one healthy subjects were divided into three gender and age-matched subgroups (mean age 58Y, 67Y and 79Y) that served as controls to THA patients over the course of the follow-up (Table 1) with age means being chosen to represent an entry, a midpoint, and an exit point. Healthy subjects and OA patients awaiting THA were recruited between 10Y and 13Y after the inclusion of THA patients had ended. Plasma was sampled from these patients and analyzed within 5 months of sampling. We did not investigate socioeconomic status. Exclusion criteria in both the THA group, the OA group awaiting THA and the healthy groups were selected to minimize the risk of interference with the validity of biomarker results. We excluded subjects in all groups suffering from any kind of malignancy, immune disorder (e.g., HIV), immune-related joint diseases (e.g., rheumatoid arthritis), or diseases affecting the bone (e.g., Paget's disease), kidneys or liver. Individuals on steroids, immunotherapy, chemotherapy, or bone-regulating drugs (e.g., bisphosphonates, denosumab, calcitonin, PTH) were excluded. In addition, healthy subjects were also excluded if they had a history of bone trauma within a year of inclusion or diseases affecting the joints (e.g., osteoarthritis). Healthy subject, OA patients awaiting THA and patients that had undergone THA were not registered as smokers although smokers in the latter group that were required to terminate smoking well ahead of surgery may have resumed their presurgery smoking habits during the follow-up.

**Table 1:** Demographic and routine laboratory data of the three groups of healthy subjects and of THA patients at inclusion. Comparisons between the three groups of healthy were made by one-way repeated measures ANOVA whereas comparison between healthy (mean age 58Y) and the THA group at inclusion (mean age 58Y) were done by Wilcoxon rank sum test. Data were partly presented in a previous publication (Cassuto et al., 2020).

Subjects	Healthy (*n* = 27)	Healthy (n = 27)	Healthy (n = 27)	THA inclusion (*n* = 24)	*P*-value comparison between healthy groups	P-value comparison of THA at inclusion vs healthy age 58Y
Age (Y) mean ± SEM (range)	58 ± 1.7 (40–68)	67 ± 0.6 (61–72)	79 ± 1 (71–88)	58 ± 1.4 (40–69)	–	–
Gender (F/M)	11/16	8/19	7/20	8/16	–	–
Height (cm)	166 ± 7.6	177 ± 1.5	174 ± 1.5	171 ± 5	0.311	0.068
Weight (kg)	77 ± 4	87 ± 2	77 ± 3	83 ± 4	0.057	0.716
BMI (kg/m^2^)	25 ± 1	27 ± 1	26 ± 0.6	26 ± 1.1	0.587	0.820
Hb	133 ± 7	144 ± 2	145 ± 3	140 ± 4	0.800	0,867
WBC (x 10^9^/L)	6.2 ± 0.4	6.3 ± 0.5	6.5 ± 0.3	6.4 ± 0.3	0.364	0.506
Platelet count (x 10^9^/L)	216 ± 17	205 ± 10	216 ± 10	209 ± 12	0.344	0.589
S-creatinine (μmol/L)	85 ± 12	90 ± 2	91 ± 5	83 ± 8	0.285	0.505

### 2.2 Radiographs

Anteroposterior pelvic radiographs plus anteroposterior and axial radiographs of the THA hip and the contralateral hip were done in the THA group and the OA group awaiting THA. Radiographs in THA patients were taken shortly before surgery (PR), on day one post-surgery (PO or 1D), 6 weeks (6 W), 3 months (3 M), 6M, one year (1Y), 2Y, 5Y, 7Y, 10Y, 13Y, 15Y and 18Y after the index operation. Radiographs of the hip included the acetabular and femoral portions of the joint with examination for joint space narrowing, subchondral lucency, marginal osteophytes, and subchondral sclerosis. In cases with radiographic signs of OA in the contralateral hip joint, the patient was excluded due to potential interference with biomarkers of the joint representing the index operation.

### 2.3 Harris hip score (HHS) and pain scores

Harris Hip Score (Harris, 1969) was measured before surgery, 1Y, 2Y, 3Y 5Y, 7Y, 10Y, 13Y, 15Y and 18Y after surgery and supplemented with a more detailed questionnaire of hip pain used to monitor subjective variables of hip function (Table 2). The HHS is a 13-item patient/clinician report of pain (44-points); function (47-points); deformity (4-points); pre- and postoperative patient reported outcome measures (PROM) (5-points). A visual analogue scale is used and then scaled to a 100-point sum (maximum perfect score = 100). Results can be interpreted with the following: <70 = poor result; 70–80 = fair, 80–90 = good, and 90–100 = excellent. Pain scores were graded as follows: 44 (no pain or negligible pain), 40 (mild pain but no functional disability), 30 (no ADL dysfunction but mild pain in connection with physical activity prompting occasional use of analgesics), 20 (ADL limited by constant moderate pain necessitating the use of analgesics on regular basis), 10 (severe pain with pronounced limitation of ADL and regular use of analgesics), 0 (severe and disabling pain at rest with continuous use of analgesics).

**Table 2:** Harris hip score (HHS) and pain scores in THA patients. PR = preoperative, 1 Year (Y) to18Y after surgery. ***p < 0.001 PR vs 1Y post-THA (Wilcoxon rank sum test).

HHS	PR	1Y	2Y	3Y	5Y	7Y	10Y	13Y	15Y	18Y
Median Range	54 25–75	94*** 65–100	97 55–100	100 70–100	96 72–100	98 51–100	100 50–100	96 84–100	100 86–100	95 89–100

Pain										
Median Range	20 0–30	44*** 20–44	44 10–44	44 20–44	44 30–44	44 30–44	44 30–44	44 30–44	44 40–44	44 44–44

### 2.4 Blood sampling and analysis of plasma biomarkers

Venous blood was drawn into EDTA tubes from THA patients one day before surgery (PR), 1D, 6W, 3M, 6M, 1Y, 2Y, 5Y, 7Y, 10Y, 13Y, 15Y and 18Y post-surgery. In the three groups of healthy controls and the group of OA patients awaiting THA, venous blood for cytokine analysis was sampled on a single occasion. Blood was sampled during normal working hours. Blood samples for cytokine analysis were centrifuged at 4 °C and immediately stored at -85 °C until analysis. Plasma biomarkers were analyzed on a high-sensitivity and wide-dynamic range platform from MesoScaleDiagnostics (Sector Imager 2400®; Rockville, Maryland, USA; for details see www.mesoscale.com). Precoated 96-well plates from MSD were used for plasma analysis of TGF-β1 (Human TGF-β1 kit, K151IUC). Matched pairs of antibodies, i.e., capture antibody (CA) and biotinylated detection antibody (DA) labeled with streptavidin SULFO-TAG®, were used for analysis of plasma biomarkers as specified below. Standard curves were created using human recombinant proteins (hRP). Plasma was mounted on uncoated standard plates from MSD (L15XA). Antibodies were used within 5 months of arrival to our lab. TGF-β2, CA: anti-human monoclonal mouse IgG2_B_ antibody (Biotechne R&D Systems, cat.no.MAB612), DA: biotinylated antigen affinity-purified polyclonal goat IgG antibody (Biotechne R&D Systems, cat.no.BAF302), hRP (Peprotech, cat.no. 100-35B). TGF-β3, CA: monoclonal mouse IgG_1_ (Biotechne R&D Systems, cat.no.MAB643), DA: biotinylated antigen affinity-purified polyclonal goat IgG antibody (Biotechne R&D Systems, cat.no.BAF243), hRP (Peprotech, cat.no. 100-36E). BMP-1, CA: monoclonal rat IgG_2B_ (Biotechne R&D Systems, cat.no.MAB1927), DA: produced in goats immunized with purified, NS0-derived, recombinant human BMP-1/PCP (procollagen C-proteinase) (aa 121–730) and subsequently biotinylated (Biotechne R&D Systems, cat.no.BAF1927), hRP (Biotechne R&D Systems, cat.no.1927-ZN). BMP-2, CA: monoclonal mouse IgG_2B_ (Biotechne R&D Systems, cat.no.MAB3551), DA: cell-derived monoclonal biotinylated anti-human antibody (Biotechne R&D Systems, cat.no.BAM3552), hRP (Prospecbio cat.no.CYT-261). BMP-3, CA: monoclonal mouse IgG_2B_ (Biotechne R&D Systems, cat.no.MAB1876), DA: biotinylated antigen affinity-purified polyclonal goat IgG antibody (Biotechne R&D Systems, cat.no.BAF113), hRP (Biotechne R&D Systems, cat.no.113-BP). BMP-4, CA: monoclonal mouse IgG_2B_ (Biotechne R&D Systems, cat.no.MAB7571), DA: monoclonal mouse IgG_1_ (Biotechne R&D Systems, cat.no.BAM7572), hRP (Prospecbio cat.no.CYT-361). BMP-6, CA: monoclonal mouse IgG_2B_ (Biotechne R&D Systems, cat.no.MAB507), DA: biotinylated antigen affinity-purified polyclonal goat IgG antibody (Biotechne R&D Systems, cat.no.BAF507), hRP (Prospecbio cat.no.CYT-754). BMP-7, CA: Antigen affinity-purified rabbit anti-human polyclonal antibody (PeproTech cat.no.500-P198), DA: Biotinylated antigen affinity-purified rabbit anti-human polyclonal antibody (PeproTech cat.no.500-P198Bt), hRP (Prospecbio cat.no.CYT-333). BMP-9, CA: monoclonal mouse IgG_2B_ (Biotechne R&D Systems, cat.no.MAB3209), DA: biotinylated antigen affinity-purified polyclonal goat IgG antibody (Biotechne R&D Systems, cat.no.BAF3209), hRP (Biotechne R&D Systems, cat.no.3209-BP). Antibodies were optimized by checkerboard titrations and subsequent control of standard curves. Inter-assay variations were <5 %.

#### 2.4.1 Stability of stored plasma

An important issue when storing blood samples over extended periods of time is the degree of degradation which could have significant impact on the interpretation of data. To ascertain the validity of stored plasma, we compared plasma levels of biomarkers in THA patients taken before surgery and stored for 18 years with levels in fresh plasma (not older than 5 months) from OA patients awaiting THA. Inter-group variability was minimized by mounting plasma from both groups on the same analytical plates. To safeguard the stability of biomarkers over the course of time, EDTA tubes containing frozen plasma (-85 °C) were thawed on ice in limited numbers before being aliquoted into cryotubes (120 μl/tube) and stored at -85 °C for short periods of time before use. Each cryotube was only thawed and used on a single occasion for analysis on four different plates (25 μl/well, see www.mesoscale.com) with excessive plasma being discarded. Evaluations at our lab showed no distinguishable changes in biomarker levels after 3 freeze/thaw cycles.

### 2.5 Statistical analysis

**Fig. 1:**
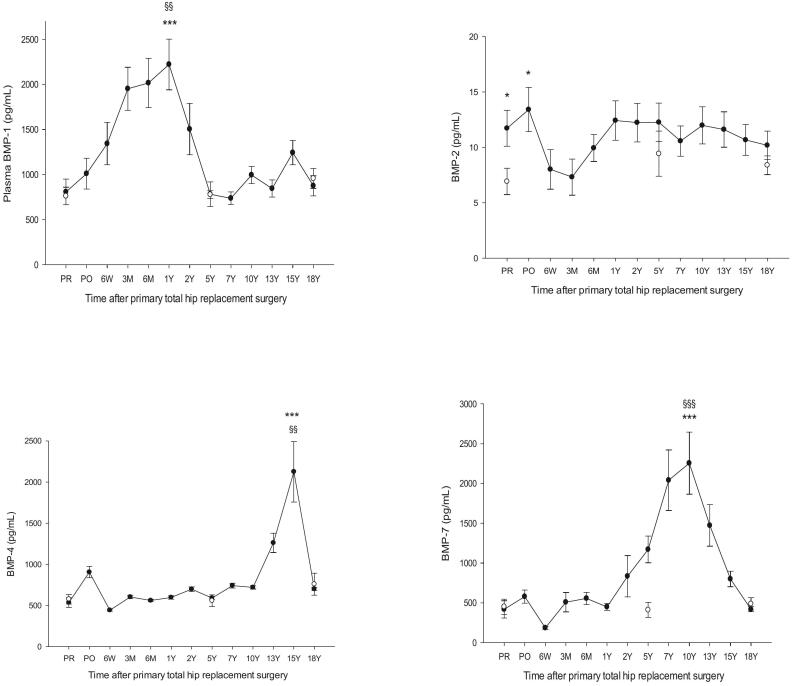
BMP-1, BMP-2, BMP-4, and BMP-7 in patients with primary total hip arthroplasty (THA) vs healthy controls. BMP-1, ***p < 0.001 THA 1Y vs healthy 58Y, §§ *p* = 0.006 THA 1Y vs THA PR. BMP-2, *p < 0.05 THA PR and THA PO vs healthy 58Y. BMP-4, ***p < 0.001 THA 15Y vs healthy 79Y, §§ *p* = 0.004 THA 15Y vs THA PR. BMP-7, ***p < 0.001 THA 10Y vs healthy 67Y, §§§p < 0.001 THA PR vs THA 10Y. Mean ± SEM.

**Fig. 2:**
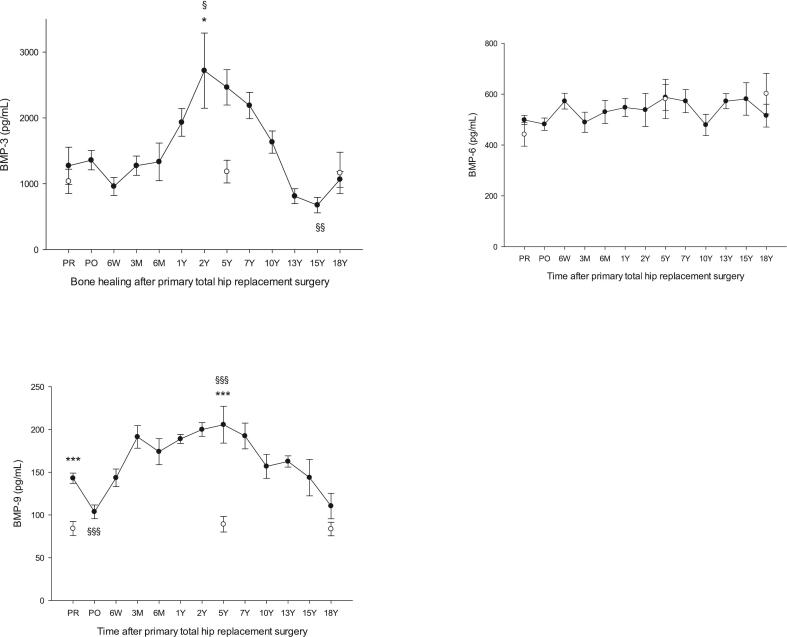
BMP-3, BMP-6 and BMP-9, in patients having undergone primary total hip arthroplasty (THA) vs healthy controls. BMP-3, *p = 0.04 THA 2Y vs healthy 58Y, ^§^*p* = 0.02 THA PR vs THA 2Y, ^§§^*p* = 0.006 THA 15Y vs THA PR. BMP-6, differences between THA and healthy or within the THA group were not significant. BMP-9, ***p < 0.001 THA PR vs healthy 58Y and THA 5Y vs healthy 67Y, ^§§§^p < 0.001 THA PR vs THA PO and THA 5Y vs THA PR. Mean ± SEM.

One-way repeated measures ANOVA with post hoc Holm-Śidak test was used to analyze differences in demographic and laboratory data (Table 1). Comparison of differences in individual biomarker levels (BMPs and TGF-βs) between individuals in the three age groups of healthy was done by means of one-way repeated measures ANOVA (Table 3). One-way analysis of variance (ANOVA) with post hoc Holm-Śidak test was used to compare biomarkers in the THA group, representing a continuous dependent variable (time), versus the independent categorical groups of healthy subjects (Fig. 1, Fig. 2, Fig. 5). Normality was assessed by Shapiro-Wilk and Kolmogorov-Smirnov tests. Equal variance was assessed by the Brown-Forsythe test. Log transformation was used to normalize data when necessary. Age-matched comparisons between the three age groups of healthy (58Y, 67Y and 79Y) and the corresponding age intervals in the THA group were performed as follows: 1) THA PR vs healthy mean age 58Y (all biomarkers), 2) THA PO, 3 M, 6 M, 1Y and 2Y vs healthy mean age 58Y (all biomarkers), 3) THA representing high activity between 5Y and 13Y vs healthy mean age 67Y and 4) THA 15Y or 18Y vs healthy mean age 79Y (all biomarkers). Age-related comparisons of biomarker levels within the THA group were done between PR (mean age 58Y) and 18Y (mean age 79Y) by Wilcoxon rank sum test. Evaluation of stability of biomarker levels in stored plasma was done by comparison of plasma sampled before surgery (stored 18Y) in the THA group (mean age 58Y) with fresh plasma from age-matched individuals in the OA group awaiting THA (mean age 58Y) by means of Wilcoxon rank sum test (Table 4). Data are presented as the mean ± SEM.

**Table 3:** TGF-βs and BMPs in the three groups of healthy controls. Mean ± SEM.

Age (Y) (range)	Healthy 58 ± 1.7 (40–68)	Healthy 67 ± 0.6 (61–72)	Healthy 79 ± 1 (71–88)	*P*-values healthy
BMP-1 (pg/ml)	761 ± 98	777 ± 43 †	956 ± 110 *§	†*§ = 0.741
BMP-2 (pg/ml)	6.9 ± 1.2	9.4 ± 2.0 †	8.4 ± 0.8 *§	†*§ = 0.305
BMP-3 (pg/ml)	1035 ± 185	1184 ± 172 †	1163 ± 314 *§	†*§ = 0.819
BMP-4 (pg/ml)	577 ± 57	558 ± 70 †	759 ± 134 *§	†*§ = 0.169
BMP-6 (pg/ml)	441 ± 46	580 ± 77 †	602 ± 79 *§	†*§ = 0.378
BMP-7 (pg/ml)	450 ± 96	410 ± 96 †	486 ± 77 *§	†*§ = 0.051
BMP-9 (pg/ml)	84 ± 8	89 ± 9 †	83 ± 7 *§	†*§ = 0.178
TGF-β1 (ng/ml)	5.8 ± 0.3	6.0 ± 0.4 †	5.0 ± 0.6 *§	†*§ = 0.303
TGF-β2 (pg/ml)	167 ± 12	168 ± 15 †	197 ± 11 *§	†*§ = 0.875
TGF-β3 (pg/ml)	237 ± 15	228 ± 13 †	259 ± 38 *§	†*§ = 0.783

Age given as mean ± SEM. One-way repeated measures ANOVA for comparison of: † Healthy 67 Y versus healthy 58 Y, ^⁎^ Healthy 79 Y versus healthy 67 Y, ^§^ Healthy 79 Y versus healthy 58 Y. †*§ Inter-group differences not significant.

**Table 4:** TGF-βs and BMPs in preoperative THA patients vs. osteoarthritis (OA) patients awaiting THA. Mean ± SEM.

Age (Y) mean ± SEM (range)	Preoperative THA 58 ± 1.7 (40–68)	Presurgery OA waiting for THA 58 ± 1 (34–86)	P-value OA vs THA
BMP-1 (pg/ml)	712 ± 86	667 ± 32	0.147
BMP-2 (pg/ml)	6.9 ± 1.2	4.3 ± 0.8	0.325
BMP-3 (pg/ml)	1159 ± 74	1170 ± 78	0.370
BMP-4 (pg/ml)	577 ± 57	535 ± 59	0.300
BMP-6 (pg/ml)	498 ± 17	460 ± 27	0.586
BMP-7 (pg/ml)	417 ± 107	450 ± 96	0.377
BMP-9 (pg/ml)	143 ± 6	176 ± 4	0.140
TGF-β1 (ng/ml)	11 ± 0.7	12 ± 0.9	0.625
TGF-β2 (pg/ml)	164 ± 7	165 ± 6	0.687
TGF-β3 (pg/ml)	202 ± 7	203 ± 7	0.132

## 3 Results

Comparison of demographic and routine laboratory data between the three groups of healthy controls and between healthy controls (mean age 58Y) and THA-patients at inclusion (mean age 58Y) were not significant (Table 1).

**Fig. 3:**
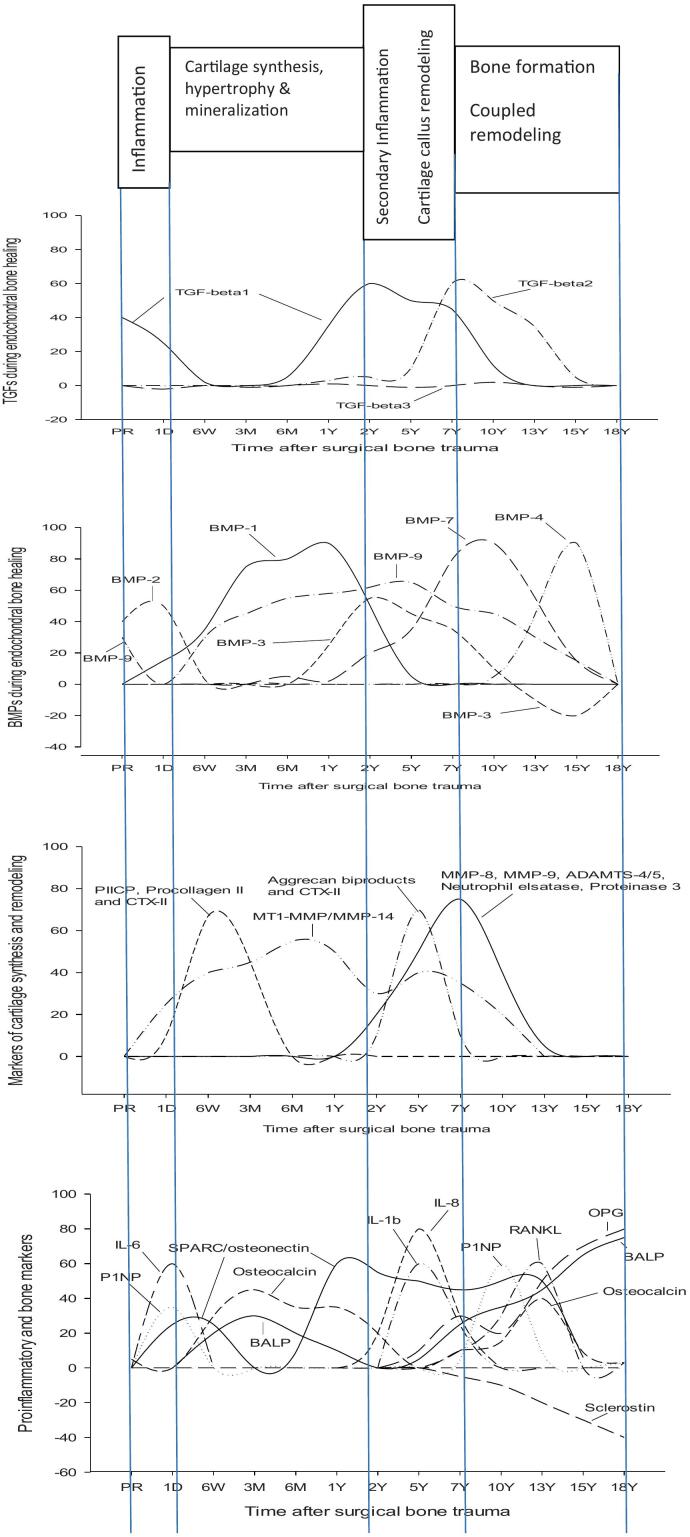
Schematic presentation of the four phases of endochondral bone repair and the spatiotemporal biomolecular responses of the current and previous studies (Cassuto et al., 2018; Cassuto et al., 2020), The upper two panels are a summary of data from the present study reflecting the results on TGF-βs (upper panel) and BMPs (second panel from top). Third panel from top is a summary of MMPs, ADAMTS and serine proteases from a previous study of the same set of patients as the current study (Cassuto et al., 2020) while the bottom panel is a summary of proinflammatory cytokines and bone markers originating from a previous study based on the same cohort of patients as the current report (Cassuto et al., 2018). Thus, all four panels originate from the same study population of uncemented total hip arthroplasty (THA) patients and healthy control subjects. Baseline (=0) is represented by levels of biomarkers in age- and gender matched healthy controls. Deviations above or below baseline represent significantly increased or decreased plasma levels in THA patients' relative healthy controls. Graph altitudes do not reflect the actual level of the individual markers as percent relative baseline. Acronyms: TGF-β (transforming growth factor beta), BMP- (bone morphogenic protein-), PIICP (procollagen II C-terminal propeptide), CTX II (C-terminal telopeptide fragments of type II collagen), MMP- (matrix metalloproteinase-), ADAMTS (a disintegrin and metalloproteinase with thrombospondin motifs), IL- (interleukin-), P1NP (N-terminal propeptide of type I collagen), BALP (bone specific alkaline phosphatase), RANKL (receptor activator of nuclear kappa B ligand), OPG (osteoprotegerin).

**Fig. 4:**
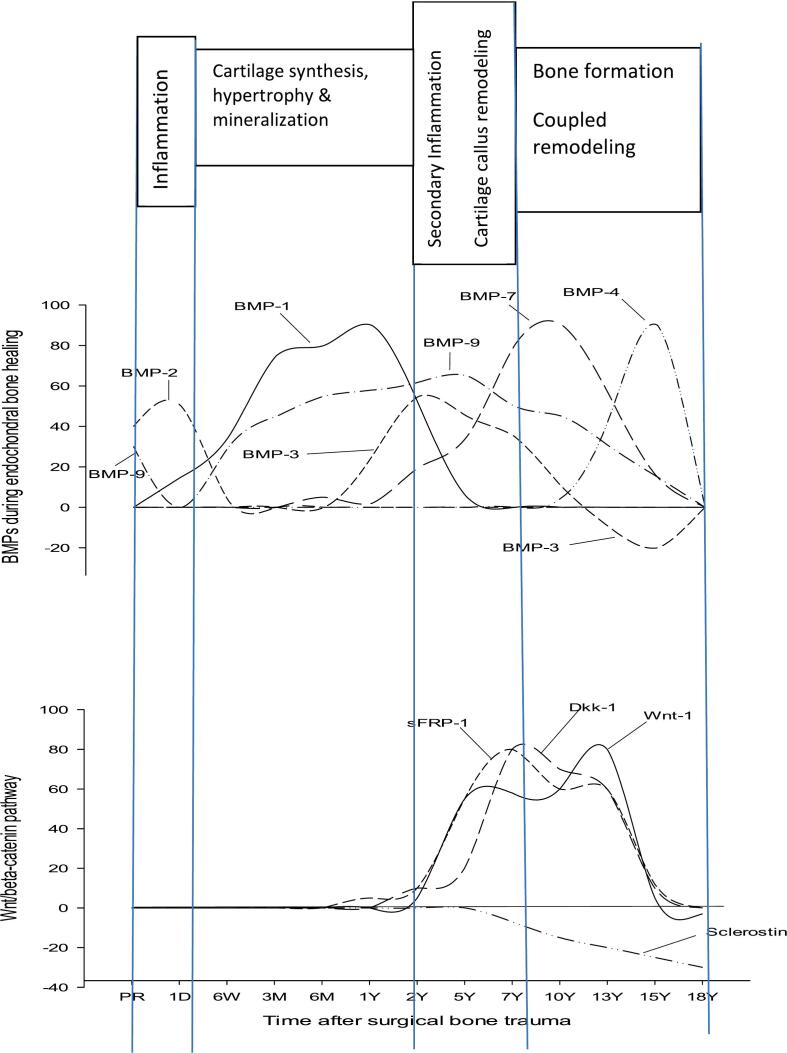
Schematic presentation of the spatiotemporal responses of BMPs (upper panel, current study) and Wnt ligands (lower panel, previous study (Cassuto et al., 2018)) during the four phases of endochondral bone repair after total hip replacement surgery. Data in the upper and lower panels are from the same population of THA patients and healthy subjects. Curves are presented as significant deviation from baseline (=healthy) but do not reflect absolute values of changes. Acronyms: BMP- (bone morphogenic protein), sFRP-1 (secreted frizzled related protein-1), Dkk-1 (Dickkopf-1), Wnt-1 (Wnt agonistic ligand-1).

**Fig. 5:**
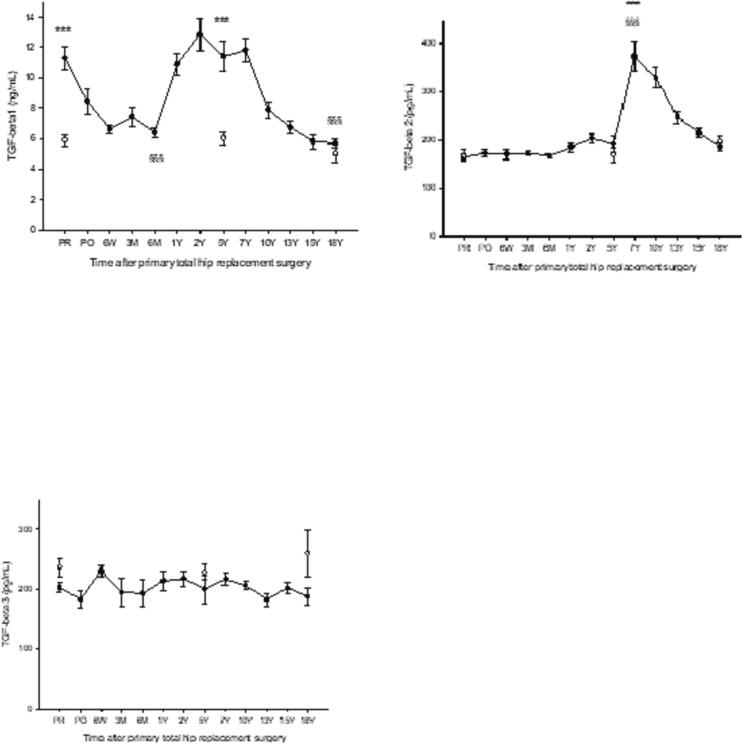
TGF-β 1, 2, 3 in patients having undergone primary total hip arthroplasty vs healthy controls. TGF-β 1, ***p < 0.001 THA PR vs healthy 58Y and THA 5Y vs healthy 67Y. ^§§§^p < 0.001 THA PR vs THA 6 M and THA PR vs THA 18Y. TGF-β 2, ***p < 0.001 THA 7Y vs healthy 67Y, ^§§§^p < 0.001 THA PR vs THA 7Y. TGF-β 3, differences between THA and healthy or within the THA group were not significant. Mean ± SEM.

HHS and pain scores are presented in Table 2. A table of HHS and pain scores from the current THA group was presented in previous studies (Cassuto et al., 2018; Cassuto et al., 2020) and shows a significant improvement in HHS and pain scores 1Y and onward relative to preoperative values. Age-related changes in biomarker levels between the three groups of healthy are presented in Table 3 and show no significant age-related differences for any of the study biomarkers. Levels of individual biomarkers in plasma of THA patients taken before surgery (PR) and stored for 18Y showed no significant differences versus the corresponding biomarker levels in fresh plasma from OA patients awaiting THA (Table 4). Fig. 1, Fig. 2, Fig. 5 show levels of study biomarkers in arthroplasty patients and healthy controls throughout the follow-up with differences between the groups depicted in the figures. Fig. 3, Fig. 4 summarize significant spatiotemporal changes of biomarkers in the context of the four phases of endochondral bone repair previously reported (Gerstenfeld et al., 2003; Loi et al., 2016; Marsell and Einhorn, 2011; Einhorn and Gerstenfeld, 2015; Bahney et al., 2019).

Results in short: BMP-1 increased immediately after surgery to reach a plateau between 3 M and 1Y before gradually returning to basal level at 5Y. BMP-2 was significantly above healthy before surgery (OA) and on day 1 post-surgery followed by a decrease to control for the remainder of the follow-up. BMP-3 was at the level of controls until 1Y post-surgery followed by a peak at 2Y with a gradual return to baseline at 10Y followed by a decrease to a nadir significantly below controls at 15Y and a subsequent return to baseline at 18Y. BMP-4 was not distinguishable from healthy controls until after 10Y postsurgery when it rose sharply to a peak at 15Y followed by a return to baseline at 18Y. BMP-6 did not deviate from healthy controls at any point during the follow-up. BMP-7 was at the level of healthy controls until 1Y post-surgery followed by a gradual increase to a peak at 10Y and a gradual decrease to baseline at 18Y. BMP-9 was above healthy before surgery with a short-lived decrease to baseline on day 1 after surgery followed by a subsequent increase to a plateau significantly above healthy and lasting from 6 W until 10Y postsurgery before gradually returning to basal level at 18Y. TGF-β1 was significantly above controls in presurgery OA patients followed by a gradual decrease to basal level at 6 W post-surgery and remaining there until 1Y before increasing to a plateau significantly above controls between 2 Y and 7 Y followed by a return to basal level at 13 Y post-surgery. TGF-β2 was at the level of controls until 5Y post-surgery after which it increased sharply to a peak at 7Y followed by a gradual return to basal level at 15Y. TGF-β3 did not differ from controls at any point during the follow-up.

## 4 Discussion

### 4.1 Osteoarthritis

BMP-2, BMP-9 and TGF-β1 in presurgery OA patients were significantly above healthy (Fig. 3). The sharp decrease in BMP-2 and BMP-9 immediately after removal of the diseased hip joint support an inductive role in OA pathology as suggested by studies showing both BMP-2 and BMP-9 to increase chondrocyte hypertrophy (Caron et al., 2013; van Caam et al., 2015), a crucial factor in OA pathology. Moreover, BMP-2 levels have been shown to be significantly elevated in the serum and joint fluids of OA patients and to positively correlate with clinical and radiographic OA severity scores (Liu et al., 2015). In similarity, TGF-β1, the predominant isoform in bone and articular cartilage, has been found at high levels in the synovial fluid of OA patients (van der Kraan, 2018) and shown in experimental studies to induce chondrocyte hypertrophy, subcondral bone resorption, osteophyte formation, synovial fibrosis and inflammation, all being hallmarks of OA joints (van der Kraan, 2018). A simple look at the negative effects of the above biomarkers on articular cartilage would suggest that they team up to exacerbate the processes that caused the pathologic appearance of OA joints in patients of the current study. Although this may apply, all three cytokines have also been shown to have positive effects on cartilage repair. Thus, BMP-2 and BMP-9 are not only promoters of the transformation of mesenchymal stem cells (MSCs) into chondrocytes but can also induce the latter's synthesis of type II collagen and aggrecan required for the maturation and maintenance of articular cartilage (Majumdar et al., 2001; Liu et al., 2018). Likewise, implantation of TGF-β1-activated scaffolds in rabbits with full-thickness cartilage defects not only increased differentiation of MSCs into chondrocytes and stimulated their secretion of type II collagen and aggrecan, but also significantly improved repair of cartilage defects (Diao et al., 2009). However, when interpreting the current results in OA patients it is important to look beyond the individual growth factors as the net outcome of their activities is subject to a complex crosstalk (Findlay and Kuliwaba, 2016). When combining BMP-2 and BMP-9 in the treatment of digit amputation in mice, Yu et al. were able to demonstrate that BMP-2 induced proliferating chondrocytes while BMP-9 stimulated joint regeneration in a synergistic way that resulted in a complete joint structure (Yu et al., 2019). In analogy, TGF-β1 was shown to block BMP-9-induced chondrocyte hypertrophy thus aiding in the preservation of a normal articular cartilage phenotype (van Caam et al., 2015). In a culture of calf metacarpal synovium incubated with BMP-2 alone, the expression of type II collagen and aggrecan increased significantly as did the gene expression levels of collagen X, a marker of chondrocyte hypertrophy and OA (Shintani et al., 2013). However, a combination of BMP-2 and TGF-β1 not only enhanced chondrogenesis and improved the properties of the neocartilage but, most importantly, reduced the overall collagen X staining equaling a suppression of the hypertrophic differentiation of chondrocytes that is coupled to mineralization and OA pathology (Shintani et al., 2013).

### 4.2 Bone healing of hip implants

Time intervals for the four phases in the current study are based on similarities with spatiotemporal responses of biomarkers that are characteristic of the various phases of endochondral repair previously described (Gerstenfeld et al., 2003; Loi et al., 2016; Marsell and Einhorn, 2011; Einhorn and Gerstenfeld, 2015; Bahney et al., 2019), i.e., Phase 1: *Primary proinflammatory phase* lasting from surgery until day 7, Phase 2: *Chondrogenic phase* from day 7 until 18 months postsurgery, Phase 3: *Secondary proinflammatory and cartilage remodeling phase* lasting from 18 months until 7Y, Phase 4: *coupled bone remodeling* from 7Y until 18Y postsurgery. The current spatiotemporal responses of BMPs and TGF-βs will be described in the context of these phases. As the current study is based on the same population of patients and controls as our previous reports (Cassuto et al., 2018; Cassuto et al., 2020), we will discuss the current results, when advantageous for clarity, against the backdrop of previous observations. In the following paragraphs, individual biomarkers will not be addressed in alphabetical order but rather in the order of spatiotemporal appearance during the process of endochondral bone repair.

#### 4.2.1 Bone morphogenic proteins

##### 4.2.1.1 BMP-2

The current study showed a significant, although short-lived, augmentation of BMP-2 immediately after THA which conforms with a previous study in mice (Cho et al., 2002). The importance of BMP-2 for endochondral repair was presented in a pivotal study (Tsuji et al., 2006) showing that mice lacking BMP-2 in the limb bones after birth have significantly reduced bone mineral density (BMD), develop weight-bearing fractures and fail to produce any callus in the fracture gap (Tsuji et al., 2006). This inability to initiate fracture repair was shown to stem from failure to start chondrocyte and osteoblast differentiation from MSCs and ignite the process of endochondral repair (Tsuji et al., 2006).

##### 4.2.1.2 BMP-1

BMP-1 increased shortly after surgery to form a plateau significantly above controls between 3 M and 1Y before gradually returning to basal level at 5Y postsurgery. This represents a significant increase by BMP-1 during the phases of MSC recruitment and condensation as well as chondrocyte proliferation and hypertrophy (Fig. 3). BMP-1 is, opposite to other BMPs, a metalloproteinase that does not belong to the TGF-β superfamily (Marsell and Einhorn, 2009). It is key to matrix formation by cleavage of the C-terminus of the major precursors of fibrillar collagens, i.e., procollagen I, II and III, in a process required for the formation of mature collagen monomers that can self-assemble into ECM fibrils of the cartilage and bone (Hopkins et al., 2007). The critical role of BMP-1 was confirmed by gene knockout studies showing failure in the normal assembly of the extracellular matrix resulting in early death due to severe anomalies of the skeleton (Hopkins et al., 2007). The conjoined increase by BMP-1 and markers of cartilage synthesis, i.e., procollagen II and PIICP (procollagen II C-terminal propeptide) and cartilage turnover, i.e., CTX II (C-terminal telopeptide fragments of type II collagen) (Fig. 3) lend further support to BMP-1 as an important factor during formation of the cartilage ECM. It is also noteworthy that SPARC (secreted protein acidic and rich in cysteine) or Osteonectin, one of the most abundant non-collagenous proteins of the ECM and expressed at high levels in cartilage and bone during formation, repair, and remodeling, peaked in parallel with BMP-1 and markers of cartilage synthesis (Fig. 3). SPARC/Osteonectin binds, in similarity with BMP-1, to procollagen I, II and III and is considered of major importance for ECM fibril formation (Bradshaw, 2009). However, while BMP-1-induced splicing of procollagens has been shown to be critical for proper collagen fibril assembly (Hopkins et al., 2007), SPARC/Osteonectin has been proposed to regulate ECM formation by attaching to the procollagens and delaying their incorporation into new fibrils until proper processing of their C-terminus by BMP-1 and N-terminus by ADAMTS-2 (a disintegrin and metalloproteinase with thrombospondin motifs-2) has been achieved (Bradshaw, 2009).

Another member of the metalloproteinase family that, in addition to BMP-1, increased during the early phase of cartilage formation in the current patient population was MT1-MMP (membrane-type 1 matrix metalloproteinase) or MMP-14 (Fig. 3). MT1-MMP belongs to a group of membrane-bound proteases with an extracellular catalytic site that, in similarity with BMP-1, is able to cleave native collagen I, II and III as well as a range of other matrix proteins (Gifford and Itoh, 2019). MT1-MMP is, like all other MT-MMPs, activated intracellularly by proprotein convertases and expressed on the cell surface as an active enzyme (Gifford and Itoh, 2019). Its importance was clearly illustrated in MT1-MMP-deficient mice showing severe anomalies of the cartilage and bone, with early death attributed to defective collagen turnover (Gifford and Itoh, 2019). Pericellular proteolysis of type I, II and III collagens by skeletal MSCs expressing MT1-MMP has been shown to be of major importance for the migration of MSCs from the bone marrow into the site of bone repair where they differentiate into chondrocytes and osteoblasts (Gifford and Itoh, 2019). The proteolytic remodeling of the ECM by MT1-MMP is required not only for the migration of chondrogenic and osteogenic cells into the ECM but also for the induction of alkaline phosphatase (ALP) and the formation.

of mineralized tissues (Alford et al., 2015). The latter agrees with our previous studies showing *bone specific* ALP (BALP) (Cassuto et al., 2018) and MT1-MMP (Cassuto et al., 2020) (Fig. 3) to jointly increase shortly after the surgical bone trauma. It is thus conceivable to assume that a crosstalk exists between BMP-1, SPARC/Osteonectin and MT1-MMT during the cartilagenous phase of endochondral repair with BMP-1 and SPARC/Osteonectin regulating the transformation of monofilaments of collagen I, II and III into the fibrillar network of the cartilage ECM (Hopkins et al., 2007; Bradshaw, 2009), whereas MT1-MMT-induced remodeling of the cartilage facilitates the migration of prechondrocytes and preosteoblasts into the ECM as well as its subsequent mineralization (Alford et al., 2015).

##### 4.2.1.3 BMP-9

The primary and short-lasting phase of augmented BMP-9 occurred immediately after the surgical bone trauma and coincided with increased levels of two metalloproteinases, i.e., BMP-1 and MT1-MMP (Cassuto et al., 2020). This would support a previously suggested role for BMP-1 and MT1-MMP in the cleavage and subsequent activation of members of the TGF-β superfamily, such as BMP-7, BMP-9, and TGF-β1 (Hopkins et al., 2007; Gifford and Itoh, 2019). The secondary phase of increased of BMP-9 was associated with augmented levels of cartilage synthesis and turnover markers (Fig. 3) which is in line with previous studies showing BMP-9 to be a key factor in endochondral cartilage formation by stimulating the chondrocyte synthesis of aggrecan and type II collagen (Majumdar et al., 2001) and by promoting cartilage hypertrophy and mineralization (van Caam et al., 2015). Interestingly, high levels of IL-1β at 5Y postsurgery (Fig. 3) did not appear to have any negative influence on the level of the prochondrogenic BMP-9 despite that IL-1β is a potent inhibitor of cartilage matrix synthesis (Majumdar et al., 2001). This agrees with previous studies showing BMP-9 to maintain its prochondrogenic activity in the presence of IL-1β (Majumdar et al., 2001) and to resist being cleaved and inactivated by the IL-1β-induced enzymes, MMP-8 and MMP-9 (Furlan et al., 2021). The importance of maintaining high BMP-9 during the proinflammatory phase of cartilage resorption and remodeling was highlighted by studies showing BMP-9 to be highly regulatory during inflammation (Song et al., 2022) and to have the ability to accelerate cartilage callus formation and remodeling (Luther et al., 2011). The role of BMP-9 is however not likely to be limited to the cartilagenous phase of endochondral repair as suggested by the current study showing its augmented trajectory to extend well beyond the cartilagenous phase and to coincide with the phases of early and late osteoblastogenesis, bone mineralization, osteoclastogenesis and coupled remodeling (Fig. 3). A bone regulatory effect is supported.

by a study in preosteoblastic cells showing BMP-9 to increase the expression of several osteoblastogenic markers through its stimulatory effect on the Wnt pathway (Zhou et al., 2020), the latter supported by the extensive crosstalk shown to exist between the Wnt and BMP pathways (Baron and Kneissel, 2013; Itasaki and Hoppler, 2010). A crosstalk between BMP-9 and Wnt ligands, such as Wnt agonistic ligand-1 (Wnt-1), frizzled-1 (sFRP-1) and dikkopf-1 (Dkk-1), previously shown to be of importance for the regulation of osteoblast and osteocyte activity during bone repair (Baron and Kneissel, 2013), is further supported by our studies showing a spatiotemporal overlap between Wnt ligands and BMP-9 during the endochondral phase of coupled remodeling (Fig. 4). Moreover, a previous study investigating the importance of the Wnt pathway for BMP-9-induced osteogenic differentiation of MSCs (Tang et al., 2009) showed that BMP-9 and the Wnt agonist, Wnt-1, enhanced each other's formation of ALP in MSCs while the Wnt antagonists, sFRP and Dkk-1, diminished BMP-9-induced ALP formation thereby reducing bone induction and mineralization (Tang et al., 2009). BMP-9-induced bone regulation is likely to also involve osteoclasts as suggested by its overlap with high levels of RANKL and OPG (Fig. 3) and supported by a study in mice showing BMP-9 to repress RANKL-induced differentiation of bone marrow macrophages into osteoclasts thereby reducing bone loss in ovariectomized mice (Zhou et al., 2020). Taken together, the current study showing significantly augmented BMP-9 levels from early chondrogenesis until the late phase of coupled bone remodeling highlights the importance of BMP-9 throughout the entire endochondral repair process and positions it in a unique spot among osteogenic BMPs.

##### 4.2.1.4 BMP-7

BMP-7 did not deviate from the level of controls until 6 M postsurgery when it gradually began to increase. As the initial increase by BMP-7 was shown to coincide with the return of cartilage synthesis markers (PIICP, procollagen II) to basal level in our patients (Fig. 3, panel 3), it is reasonable to assume that the involvement of BMP-7 in chondrogenesis, although previously shown to promote chondrogenic matrix synthesis (Caron et al., 2013), is set to begin when fibrillar assembly and organization of the cartilage is at advanced stage under the influence of mediators, such as BMP-1 (Hopkins et al., 2007), SPARC/Osteonectin (Bradshaw, 2009), MT1-MMP (Gifford and Itoh, 2019) and BMP-9 (Tang et al., 2009) (Fig. 3). In the following, BMP-7 increased to a peak 7-10Y after THA which coincided with high levels of proinflammatory cytokines (IL-1β, IL-8) and enzymes (MMPs, ADAMTS, serine proteases) (Fig. 3), all being part of the processes that transform the cartilage into bone. The involvement of BMP-7 in cartilage remodeling is further supported by a recent study showing BMP-7 to upregulate the expression of MMP-8, MMP-9 and MMP-13 in fibroblasts which in turn increases the enzymatic release and activation of BMP-7 and triggers a positive loop of mutual activation between BMP-7 and MMPs (Furlan et al., 2021). In addition to MMPs, the peak of BMP-7 was shown to coincide with peak levels of ADAMTS 4 and ADAMTS 5, another family of enzymes involved in the degradation and remodeling of the cartilagenous callus (Kelwick et al., 2015) (Fig. 3). However, in contrast to the stimulatory effect of BMP-7 on MMPs, its effect on ADAMTS 4/5 is inhibitory (Wang et al., 2014). This moderation of enzymatic activity by BMP-7 during the cartilage remodeling phase is likely to be of importance for a gradual and orderly transition of the cartilage into bone, a role supported by studies showing BMP-7 to suppress chondrocyte hypertrophy and calcification (Caron et al., 2013), a prerequisite for enzymatic degradation of the hypertrophic cartilage before being transformed into bone (Gerstenfeld et al., 2003). Another important regulatory path assigned to BMP-7 is derived from its potent inhibition of proinflammatory cytokines (Gould et al., 2002), as suggested by it coinciding with IL-1β and IL-8 in the current THA population (Fig. 3). This appears to be part of a general anti-inflammatory effect attributed to BMP-7 and characterized by redirection of M1 macrophages responsible for the synthesis of proinflammatory cytokines towards M2 macrophages that produce anti-inflammatory cytokines that contribute to the resolution of inflammation (Singla et al., 2016), a key factor for bone repair to proceed normally (Newman et al., 2021). The process of replacing cartilage with bone is characterized by MSCs being transformed into osteoblasts that fill the empty lacunae left behind by apoptotic chondrocytes (Gerstenfeld et al., 2003). Our study showing augmented BMP-7 to coincide with markers of osteoblast differentiation, i.e., type I collagen (P1NP) and BALP (Fig. 3), increases the likelihood that BMP-7, a potent inducer of osteoblastic differentiation (Lavery et al., 2009), is part of the osteogenesis process. A crosstalk between BMP-7 and another important osteogenic factor, namely BMP-9, is suggested by their significant overlap during the osteoblastogenic phase (Fig. 3). Notwithstanding that the two BMPs engage different sets of receptors and are regulated differently by endogenous inhibitors (Gipson et al., 2020), they share several common properties of importance for endochondral repair, such as chondrogenesis (Caron et al., 2013), regulation of inflammation (Song et al., 2022; Gould et al., 2002; Singla et al., 2016), osteogenesis (Luther et al., 2011; Lavery et al., 2009; Wang et al., 2017) and osteoclastogenesis (Lowery and Rosen, 2018) while at the same time having opposing actions on the formation and mineralization of the hypertrophic cartilage (Caron et al., 2013; van Caam et al., 2015). This suggests that, although BMP-7 and BMP-9 induce their osteogenic actions in part via distinct mechanisms (Gipson et al., 2020), they are likely to have redundant and compensatory osteogenic effects that enable bone repair to proceed despite imbalance in the level of the individual BMPs (Lowery and Rosen, 2018). Moreover, both BMPs showed a significant spatiotemporal overlap with agonistic and antagonistic ligands of the bone anabolic Wnt pathway (Fig. 4) which lends support to previous studies showing BMP-7 (Krause et al., 2010) and BMP-9 (Luther et al., 2011; Tang et al., 2009) to exert their osteogenic effects in part through a complex crosstalk with the Wnt signaling pathway (Baron and Kneissel, 2013; Lowery and Rosen, 2018; Kamiya and Mishina, 2011). The latter is further supported by a recent study (Zhang et al., 2022) showing that activation of Wnt signaling in osteocytes significantly increased their BMP-7 expression as well as the induction of bone by MSCs.

##### 4.2.1.5 BMP-3

BMP-3 is a monomer that lacks the seventh cysteine residue of other BMPs and can therefore not dimerize which renders it a non-signaling decoy ligand that blocks the activin receptor II (*Act*RII), a common receptor of several structurally related members of the TGF-β superfamily such as, Activin, BMP-2, BMP-4 and BMP-7 (Lowery and Rosen, 2018; Gamer et al., 2005). As signal transduction by Activin requires the ligand to form a complex with *Act*RI and *Act*RII, its activity is entirely blocked by BMP-3 whereas BMP-2, BMP-4 and BMP-7, which in addition to *Act*RII/BMPRI, can signal through a complex of BMPRI/BMPRII, are regulated rather than blocked by BMP-3 (Gamer et al., 2005). BMP-3 was at control level from presurgery until 6 M after surgery before rising to a peak at 2Y that coincided with chondrocyte hypertrophy and mineralization (Fig. 3). The latter agrees with a study of the growth plate showing high expression of BMP-3 in hypertrophic chondrocytes (Banovac et al., 2022). The importance of increased BMP-3 during cartilage hypertrophy, mineralization and remodeling was illustrated by a study in BMP-3 KO mice showing negligible degree of cartilage mineralization and, consequentially, delayed bone formation and bridging of the growth plate (Banovac et al., 2022). Although BMP-3 KO mice show no specific skeletal phenotype relative wild-type (WT) littermates during developmental and neonatal periods, they exhibit significantly higher bone volume during adulthood (Lowery and Rosen, 2018). The latter supports BMP-3 as a negative regulator of bone formation by blocking *Act*RII and suppressing the differentiation of MSCs into osteoblasts (Lowery and Rosen, 2018; Gamer et al., 2005). The negative regulatory effect of BMP-3 on early osteoblastogenesis could, at least in part, be mediated through inhibition of the pro-osteoblastogenic BMP-7 with which it shares the *Act*RII receptor (Lowery and Rosen, 2018; Gamer et al., 2005). Such interaction is supported by the current study showing the peak of BMP-3 to overlap with that of BMP-7 during the phase of osteoblast differentiation as shown by increased levels of the osteoblast synthesis markers P1NP, BALP and osteocalcin (Fig. 3), and markers of the bone anabolic Wnt system (Fig. 4). A crosstalk between BMP-3 and Wnt is in agreement with a study showing the Wnt agonist, Wnt-1, to induce BMP-3 expression in osteoblasts and the Wnt antagonist, Dkk-1, to reduce it (Kokabu and Rosen, 2018). A noteworthy observation of the current study was that the BMP-3 curve, being *positive* during the late chondrogenic and early osteogenic phase, was inverted and became *negative* during the phase of coupled bone remodeling (Fig. 3). Although osteoclast numbers are not affected in BMP-3 KO mice (Daluiski et al., 2001), an effect on osteoclast function cannot be ruled out. The current study showing negative BMP-3 levels to coincide with peak levels of BMP-4 and RANKL would support a dual regulatory role on osteoclasts. On the one hand, suppressed BMP-3 would alleviate the *Act*RII-induced inhibition of BMP-4 and allow the latter to increase its stimulatory effect on osteoclasts (Kamiya and Mishina, 2011) while, on the other hand, it would reduce the BMP-3-induced inhibition of Activin (Gamer et al., 2005) and increase the latter's dominant inhibition of RANKL-induced osteoclast differentiation and activity (Fowler et al., 2015). Thus, despite being a non-signaling decoy ligand, the importance of BMP-3 for the regulation of bone metabolism is supported by its abundance in bone and by studies showing it to be an important promoter of hypertrophic cartilage mineralization, a negative regulator of osteoblast formation and a dual regulator of osteoclast activity.

##### 4.2.1.6 BMP-4

A highly significant and narrow spatiotemporal peak at around 15Y post-surgery suggests that BMP-4 is primarily involved in the late phase of coupled bone remodeling characterized by extensive regulatory interaction between osteoblasts, osteocytes and osteoclasts with the aim of fine-tuning bone repair activity and maintaining bone homeostasis (Gerstenfeld et al., 2003). Mice with osteoblast-specific overexpression of BMP-4 have been shown to develop severe osteopenia due to increased numbers of osteoclasts, whereas mice overexpressing Noggin, a BMP-4 antagonist, have shown increased bone volume due to reduced numbers of osteoclasts (Kamiya and Mishina, 2011). The potent osteoclastogenic effect of BMP-4 was further supported by studies showing that conditional disruption of the BMPR1a receptor in osteoblasts, for which BMP-4 is a potent ligand, decreased the RANKL/OPG-ratio and consequentially reduced osteoclast formation leading to increased bone mass (Kamiya and Mishina, 2011; Heubel and Nohe, 2021). The current study showing the initial phase of increased BMP-4 to coincide with high levels of RANKL (Fig. 3) would support a BMPR1a-mediated pro-osteoclastogenic effect by BMP-4. However, the spatiotemporal peak of BMP-4 was also shown to coincide with markers of bone formation and mineralization, i.e., Osteocalcin, OPG, BALP and Sclerostin (Fig. 3, Fig. 4), and to overlap with the primarily pro-osteoblastogenic BMP-7 and BMP-9, which could suggest a counter-balancing role for BMP-4 during the coupled bone remodeling phase. Sclerostin has a particularly interesting role in the current context as it, besides being a well-established and potent inhibitor of the bone anabolic Wnt system (Baron and Kneissel, 2013), is also a potent inhibitor of both BMP-4 and BMP-7 (Winkler et al., 2003). An increasingly suppressed Sclerostin during the late phase of coupled bone remodeling (Fig. 4) would, on the one hand, reduce the inhibition of the pro-osteoclastogenic BMP-4 and, on the other hand, lessen the inhibition of the pro-osteoblastogenic BMP-7 (Winkler et al., 2003) and Wnt pathway (Baron and Kneissel, 2013) thereby accelerating bone turnover and improving bone quality and strength (Kamiya and Mishina, 2011). In two important previous studies the authors were able to show that a conditional deletion of BMP-4 (Tsuji et al., 2008) or BMP-7 (Tsuji et al., 2010) failed to interfere with normal fracture repair, whereas a combined deletion of BMP-4 and BMP-7 severely compromised the skeleton after birth (Tsuji et al., 2010). When projecting the latter studies (Tsuji et al., 2008; Tsuji et al., 2010) on the current results it becomes obvious that, although individual BMPs have distinct spatiotemporal patterns and a unique role to play during endochondral repair, they also show various degrees of overlap (Fig. 3&4) and functional redundancies (Lowery and Rosen, 2018) that explain their far reaching compensatory abilities (Tsuji et al., 2008; Tsuji et al., 2010).

#### 4.2.2 Transforming growth factor-βs

##### 4.2.2.1 TGF-β1

The current results are in line with previous experimental studies showing a biphasic post-fracture response by TGF-β1 characterized by a primary peak within 24 h of the bone trauma and a secondary peak during the early preosteoblastic phase of endochondral ossification (Barnes et al., 1999). A similar bi-phased response by TGF-β1 was shown in a study of patients with pseudarthrosis of the long bones characterized by high TGF-β1 level at inclusion, representative of instability at the fracture site, followed by a gradual decrease following stabilization and a renewed increase in patients shown to heal on radiographs vs. non-healing counterparts (Chaverri et al., 2022). Endochondral bone formation begins with recruitment and migration of prechondrogenic mesenchymal cells into the damaged area where they condensate and produce a matrix rich in type I and type II collagen (Goldring et al., 2006). TGF-β1 is one of the earliest signs of prechondrogenic cell migration and condensation (Goldring et al., 2006) and its importance for the latter process was demonstrated by severely undermined collagen maturation and matrix deposition in TGF-β1 KO mice (Janssens et al., 2005). TGF-β1-induced cell migration was previously shown to be blocked by a wide-range MMP inhibitor (Seomun et al., 2008) thus confirming the crucial role of MMP-induced degradation and remodeling of the basement membrane and ECM for cell migration into the area of injury (Cassuto et al., 2020). A conjoined increase by TGF-β1 and MT1-MMP (MMP-14) (Fig. 3) immediately after the surgical bone trauma would support a crosstalk during the aforementioned cell migration and be in line with a previous study showing inhibition of MT1-MMP to block TGF-β1-induced cell migration (Seomun et al., 2008). The primary peak of TGF-β1 decreased to the level of healthy individuals at 6 W postsurgery thus coinciding with the peak of type II collagen synthesis (Fig. 3) thus concluding the role of the initial peak which is in accordance with a previous study showing the primary peak of TGF-β1 to decrease to a low point in connection with the peak of type II collagen expression (Barnes et al., 1999). The secondary peak of TGF-β1 coincided with the formation of the hypertrophic cartilage (Fig. 3) which may be related to a study showing subcutaneous injection of TGF-β1 in mice to induce hypertrophic bone-like tissue (Futrega et al., 2021). The secondary peak of TGF-β1 did also coincide with the secondary proinflammatory and cartilage remodeling phase at 5–7 Y post-surgery characterized by high levels of IL-1β and IL-8 (Fig. 3). TGF-β1 is, in similarity with proinflammatory cytokines, a potent chemotactic factor (Brandes et al., 1991) that recruits neutrophils to the site of bone injury where they release proteases that digest the mineralized avascular hypertrophic cartilage and enable the.

ingrowth of blood vessels, a prerequisite for its remodeling into bone. A noteworthy observation was that the primary and secondary peaks of TGF-β1 were shown to coincide with the two peaks of MMP-9 (Fig. 3) which would support a previous study showing MMP-9 to be an important activator and regulator of TGF-β1 (Yu and Stamenkovic, 2000). Another notable observation was that the secondary peaks of TGF-β1 and MMP-9 (Fig. 3) were closely aligned with the spatiotemporal response of VEGF-A (to be published) in the current population of hip arthroplasty patients. This agrees with studies showing both MMP-9 and TGF-β1 to induce angiogenesis, the former through proteolytic release and activation of VEGF from the cartilage matrix (Colnot et al., 2003) and the latter by acting on VEGF-receptors on endothelial cells (Ferrari et al., 2009). The conjoined increases by MT1-MMP, MMP-8, MMP-9, MMP-13 and TGF-β1 during cartilage hypertrophy and remodeling in our studies (Fig. 3) is also in line with previous studies showing a perpetual and reciprocal crosstalk to exist between MMPs and TGF-β1 that is likely to be part of the mechanisms that regulate angiogenesis and cartilage remodeling during endochondral repair (Ortega et al., 2004). A crosstalk between TGF-β1 and osteogenic BMPs does also fit into the current results by showing augmented levels.

of TGF-β1, BMP-7 and BMP-9 to coincide during the early osteoblastogenic phase characterized by markers of osteoblast differentiation (BALP, type I collagen) (Fig. 3). Previous studies have shown TGF-β1 alone to be unable to induce differentiation of MSCs into osteoblasts whereas BMP-9 alone is capable of such transformation (Li et al., 2015). However, when combined, TGF-β1 will potentiate BMP-9-induced osteoblastic formation and ALP synthesis (Li et al., 2015). In contrast, the effect TGF-β1 on BMP-7 is inhibitory (Ehnert et al., 2012) which could suggest that TGF-β1 functions as a balancing factor during the recruitment and commitment phases of osteoblasts.

##### 4.2.2.2 TGF-β2

TGF-β2 remained at the level of healthy during the entire phase of cartilage formation, hypertrophy, and remodeling (Fig. 3) before increasing sharply at 5Y postsurgery and reaching a peak between 7Y and 10Y in conjunction with the peak of neutrophil-derived enzymes responsible for cartilage degradation and remodeling (Fig. 3). A similar delay was shown in a study of pseudarthrosis patients treated surgically with a bone graft or MSCs (Chaverri et al., 2022) and showing plasma TGF-β2 to remain at presurgery level until 12 months postsurgery before showing a significant increase. The involvement of TGF-β2 in neutrophil-induced remodeling of the cartilage is supported by a study showing a perpetual cycle to exist between TGF-β2 and neutrophils, with the former triggering neutrophil chemotaxis and the latter synthesizing and releasing TGF-β2 from the ECM (Szymkowiak et al., 2000). In further support, TGF-β2 has been shown to induce cartilage degradation when injected into rabbits (Elford et al., 1992). The current study also shows TGF-β2 to be significantly elevated during the ensuing phase of osteoblast differentiation characterized by augmented levels of type I collagen (P1NP) and BALP (Fig. 3) which would lend support to a stimulatory effect on osteoblastic differentiation from osteoprogenitor cells (Erlebacher et al., 1998). Notably, the trajectory of TGF-β2 extended well beyond that of TGF-β1 to also include the phase of coupled remodeling (Fig. 3) which conforms with a study in mice overexpressing TGF-β2 and showing increased rates of bone matrix turnover (Erlebacher et al., 1998). In support, TGF-β2 KO mice were shown to have aberrant skeletogenesis that primarily affected the long bones that are formed by endochondral ossification (Sanford et al., 1997) thus supporting TGF-β2 as the most likely candidate among the TGF-β isoforms to be of major importance for coupled bone remodeling during endochondral bone repair.

## 5 Clinical and practical applications

The use of antibodies to document the complexity of bone healing by means of cytological, experimental and clinical studies has been ongoing for several decades and has significantly increased our understanding of the detailed mechanisms that regulate the healing process (Gerstenfeld et al., 2003; Loi et al., 2016; Marsell and Einhorn, 2011; Einhorn and Gerstenfeld, 2015; Bahney et al., 2019). The aim of the latter research has been to describe and understand the biomolecular responses to bone trauma, be they accidental or surgical, and the subsequent mechanisms that are triggered by the bone to restore it to the original shape and function. The current study along with two previous reports emanating from the current population of hip arthroplasty patients (Cassuto et al., 2018; Cassuto et al., 2020) enabled us to describe the spatiotemporal responses of over 50 biomarkers during a two-decade follow-up that culminated in the final osseointegration of the implants. We found that the responses of biomarkers during the process of osseointegration were very similar to those seen during experimental studies of normal endochondral fracture repair (Gerstenfeld et al., 2003; Loi et al., 2016; Marsell and Einhorn, 2011; Einhorn and Gerstenfeld, 2015; Bahney et al., 2019), except that the four phases of bone repair in THA patients were extended over a significantly longer period of time (Cassuto et al., 2018; Cassuto et al., 2020). This may seem intriguing and does not conform with a prevailing view that osseointegration of hip implants reaches a final phase of bone healing within 2 years of surgery. The latter view is primarily based on perceived early stiffness of periimplant tissues and radiographic evidence of calcification around the implant shaft. However, our studies show that the phase from surgery until 2Y postsurgery is biologically characterized by the exclusive formation of cartilage that is gradually being calcified (Fig. 3) which would explain early stiffness and calcification seen on radiographs. When considering that transformation of the calcified cartilage into woven bone is a continuous and seamless process by which apoptotic chondrocytes in the collagenous matrix are being replaced by osteoblasts (Shapiro and Wu, 2019), it is understandable that it is difficult to separate the calcified fibrocartilage from woven bone on plain radiographs. There are several factors that could explain the slow progress of bone repair in implant patients, with the most recognized being: 1) excessive micromotion between the implant and surrounding bone during weight bearing 2) material compositions and their mechanical properties 3) bioactive coatings and 4) toxic debris produced from the implants (Grzeskowiak et al., 2020). Host-related factors such as age, nutrition, medications, diseases and smoking that affect the bone healing process could also contribute to a delayed (or derailed) healing process (Grzeskowiak et al., 2020). However, we believe that the most important reason for the significantly extended process of bone repair shown in our studies is that a foreign body, the shaft, is inserted into a highly immune-active site of bone repair, i.e., the femoral cavity, with all the consequences this could have on the healing process (Christo et al., 2015). Despite all the implant- and host-related factors shown to have importance for osseointegration of implants (Grzeskowiak et al., 2020; Christo et al., 2015), it is an undisputable fact that a vast majority of hip implants can overcome these hurdles and osseointegrate successfully. This leads us to the importance of the current and preceding studies (Cassuto et al., 2018; Cassuto et al., 2020) showing that bone healing of hip implants follows a predetermined path similar to previously documented spatiotemporal phases of normal endochondral bone repair (Gerstenfeld et al., 2003; Loi et al., 2016; Marsell and Einhorn, 2011; Einhorn and Gerstenfeld, 2015; Bahney et al., 2019). In addition to increased knowledge and understanding of the mechanisms that regulate normal fracture repair, the current studies shed important and critical light on the biomolecular mechanisms that govern successful osseointegration of hip implants. This is particularly important for the understanding and identification of the role of specific biomolecules in the processes that cause an implant to fail during its lengthy healing. As our lab possesses blood samples from several other longitudinal THA studies, we have been able to gradually analyze biomarkers from accumulating numbers of osteolysis patients which will enable us to investigate differences in the biomolecular patterns of these patients relative to the current successfully integrated implants. This knowledge will contribute to the identification of important mechanisms of implant failure and be a useful aid in the research and development of new drugs that target specific biomarkers (proteins) that have been identified as disease-driving factors (Shepard et al., 2017). A significant transformation in drug development has taken shape during the two most recent decades which has led to the introduction of increasing numbers of “biological” drugs based on monoclonal antibodies (mAbs) and related proteins that have revolutionized the treatment of a significant number of human diseases and conditions, some related to the bone and cartilage (Shepard et al., 2017). The use of biological drugs to treat bone related diseases in humans is based on basal and clinical research using specific antibodies that identify the role of a vast number of proteins that control all stages of normal bone and cartilage repair as well as disease-driven conditions (Meng et al., 2022). BMPs have been acknowledged as significantly osseoinductive with two being approved by the federal drug administration (FDA) for human therapy in bone regeneration, i.e., BMP-2 and BMP-7, although the latter has been taken off the market recently (Gillman and Jayasuriya, 2021). As the population of the western hemisphere grows older, significant numbers of individuals receive hip implants. Many of these ageing individuals are also prone to be treated for osteoporosis by use of e.g., Prolia® (denosumab), a blocker of the osteoclast promoter, RANKL. The use of a RANKL-blocker during the second decade of implant osseointegration could thus be problematic as it would interfere with an important part of bone maturity during the coupling phase. Thus, knowledge of what to expect during the healing process would undoubtedly facilitate the use or disuse of drugs that could support or interfere with bone repair as new biological drugs that target specific biomarkers are continuously emerging.

### 5.1 Limitations

There are limitations to the current study. One is that only limited demographic and drug use data are reported in the THA and healthy cohorts. Moreover, we did not report or exclude patients on drugs other than those known to affect bone metabolism, such as steroids, immunotherapy, or bone-regulating drugs (e.g., bisphosphonates, denosumab, calcitonin, PTH). Three patients on prohibited drugs (bisphosphonates) were identified, albeit excluded on other criteria. Aside from a history of recent bone trauma, no explicit medical and drug use data, other than that being used for exclusion of THA patients, were considered when recruiting healthy subjects. Second, we measured biomarkers in plasma that do not only reflect changes related to the implant as they may also originate from other parts of the musculoskeletal system. We believe however that the latter changes would be equally reflected in the population of gender/age-matched healthy controls. Third, recruitment of healthy controls and OA patients awaiting THA was done 10Y and 13Y into the study. A particular strength of the study is that biomarkers were sampled for two decades in a single population of arthroplasty patients and analyzed on a high-sensitivity and wide dynamic range platform allowing for detection of minor changes with high accuracy.

## CRediT authorship contribution statement

**Jean Cassuto:** Writing – review & editing, Writing – original draft, Visualization, Validation, Supervision, Software, Resources, Project administration, Methodology, Investigation, Funding acquisition, Formal analysis, Data curation, Conceptualization. **Agnetha Folestad:** Writing – review & editing. **Jan Göthlin:** Writing – review & editing, Visualization, Methodology, Investigation. **Henrik Malchau:** Writing – review & editing, Methodology, Investigation, Data curation, Conceptualization. **Johan Kärrholm:** Writing – review & editing, Resources, Project administration, Methodology, Investigation, Funding acquisition, Formal analysis, Data curation, Conceptualization.

## Declaration of competing interest

None.

## Data Availability

Data will be made available on request.
